# Promotion in the R.A.M.C.

**Published:** 1918-05-04

**Authors:** 


### PROMOTION IN THE R.A.M.C.

In a recent issue of the *Times* Mr. A. O. Warren criticises certain promotions to the Major-General's list. He claims to be the mouthpiece of the dissatisfied and presumably the disappointed, and only considers one side of the case. There is an element of truth in the letter. It is a hard fact that devotion to the careful study of some one set of scientific problems, such as those of bacteriology, hygiene, and tropical diseases, is not a preparation for administrative work. The problems are so absorbing, so important, that he who devotes himself to them is apt to let the exacting work he is doing take up all his time and prevent him from attaining all that varied knowledge of men, customs, regulations, and disciplinary matters that is necessary to form a first-class administrative officer. This is not necessarily the case, nor is it the case that if a man is a good administrator he will be passed over because he is a first-class scientific man. But it is the case that devotion to science may lead to failure to develop administrative ability.

Since it would not be right to pitchfork men who cannot administer into administrative billets as a reward for scientific work, and since it is not right nor expedient that devotion to science should come to be considered by the devotee a bar to advancement, measures should be taken to secure to him who gives himself up to pure science chances of reward equal to or greater than those that may come to him who has proved himself suited to administrative office—chances may be found in the Army Service, such appointments as professorships at Millbank, or may be found outside the Army Service by giving to Army men of proved scientific attainments appointments in civil life, such as exist now, and such as may be multiplied under the Ministry of Health that is to be formed. It is in the air that science is to take a better place in our comity after the war, and that the rewards

**PROMOTION IN THE R.A.M.C.**—*(continued)*.

for the men with knowledge are to be greater than they have been. The Army man should share in the honours and pecuniary advantages.

The last paragraph of the letter implies jobbery. Such accusations are usually vague. Irishmen always hang together, and their record is such as makes men suspicious of Irishmen's fairness to men of other nationalities and to the Protestant in religion. This suspicion is at the root of the Ulstermen's opposition and the Protestant settlers' fear of Home Rule. But it must be remembered that, at the time when the men who have been promoted entered the Army, the English schools were holding back from the Service, and were seldom represented by good men, and that the majority of officers entering the Army Medical Service came from Irish schools. This probably explains a set of promotions that, on casual inspection, might be interpreted as a piece of jobbery. Since for twenty odd years the Medical Service of the Army has been steadily improving and annually drawing to itself a first-class set of men from the great London schools, it is very unlikely in the future to happen that 90 per cent. of promotions to Major-General shall be of Irish nationality.

